# Lifestyle Factors in the Association of Shift Work and Depression and Anxiety

**DOI:** 10.1001/jamanetworkopen.2023.28798

**Published:** 2023-08-14

**Authors:** Minzhi Xu, Xiaoxv Yin, Yanhong Gong

**Affiliations:** 1 Department of Social Medicine and Health Management, School of Public Health, Tongji Medical College, Huazhong University of Science and Technology, Wuhan, Hubei, China

## Abstract

**IMPORTANCE:**

Shift work may lead to adverse health outcomes. Whether shift work is associated with depression and anxiety, and to what extent lifestyle mediates the associations, remains unknown.

**OBJECTIVE:**

To explore the associations of shift work, its type, frequency, and working years with anxiety and depression and to examine the potential mediating role of lifestyles.

**Design, Setting, and Participants:**

This cohort study included 175 543 employed or self-employed workers who participated in the UK Biobank baseline survey (2006-2010). Data analysis was conducted from November 2022 to January 2023.

**EXPOSURES:**

Employment and shift work status information was obtained from baseline. Lifestyles included smoking, physical activity, alcohol consumption, dietary characteristics, sleep duration, sedentary time, and body mass index (BMI).

**MAIN OUTCOMES AND MEASURES:**

Depression and anxiety were identified based on electronic health records. Cox proportional hazard regression models were used to calculate the association of shift work with anxiety and depression, and cause mediation analyses we used to test the mediating role of lifestyle factors in this association.

**RESULTS:**

Of the 175 543 included participants (mean [SD] age, 52.6 [7.1] years; 88 290 men [50.3%]; 167 495 White participants [95.4%]), 27 637 participants (16.2%) reported shift work. During a median (IQR) follow-up of 9.06 (8.35-9.75) years, 3956 workers (2.3%) developed depression and 2838 (1.7%) developed anxiety. In the fully adjusted model, individuals who reported engaging in shift work, or shift workers, had a higher risk of depression (HR, 1.22; 95% CI, 1.12-1.33; *P* < .001) and anxiety (HR, 1.16; 95% CI, 1.04-1.28; *P* < .001), and the risk was positively associated with shift frequency. Among shift workers, there was no significant difference between night shifts and nonnight shifts. In the dose-association analyses, years of shift work were negatively associated with the risk of depression and anxiety. Smoking, sedentary time, BMI, and sleep duration were identified as the main potentially modifiable mediators. These mediators together explained 31.3% of the association between shift work and depression and 21.2% of the association between shift work and anxiety.

**CONCLUSIONS AND RELEVANCE:**

In this cohort study, shift work was significantly associated with a higher risk of depression and anxiety, and lifestyle factors partially mediated the associations. These findings not only support that shift work should be considered an occupational hazard, but also provide evidence for the urgent need for the development of public health interventions that promote healthy lifestyles aimed at improving the mental health of shift workers.

## Key Points

**Question:**

Is shift work associated with depression and anxiety, and if so, does lifestyle mediate the associations?

**Findings:**

In this cohort study of 175 543 participants, shift work was significantly associated with a higher risk of depression and anxiety, and lifestyle factors partially mediated the associations. These mediators together explained 31.3% of the association between shift work and depression and 21.2% of the association between shift work and anxiety.

**Meaning:**

These findings suggest that the development of public health interventions related to the promotion of healthy lifestyles may improve the mental health of individuals who reported shift work.

## Teaser

This cohort study examines the association of shift work, its type, frequency, and working years with anxiety and depression to examine the potential mediating role of lifestyle factors.

## Introduction

Health and happiness are the long-standing pursuit of human beings. However, with increasing competition in society, the number of people who are experiencing mental disorders is increasing dramatically.^1^ Depression and anxiety, which reportedly affect 322 million and 264 million people worldwide, have become the leading causes of disability and premature death worldwide.^2^ Therefore, the identification and control of risk factors are critical for primary and secondary prevention of depression and anxiety.

Due to the development of advanced service cultures, shift work (ie, work arrangements outside normal working hours, including in the afternoon, evening, or rotating within these types of shifts) is becoming more common.^3,4,5^ Evidence shows that long-term shift work may lead to various diseases, such as cardiovascular diseases and mental disorders, which has triggered extensive discussions in the society on the development of health protection measures for individuals who report shift work, or shift workers.^6,7,8,9,10^ The mechanisms by which shift work contributes to cardiovascular disease have been extensively studied, however, important gaps remain regarding the association and pathways of shift work with depression and anxiety.^3,11^ Much of the current evidence ignores the potential role of shift work type, frequency, and working years, and misses job-related confounding factors, such as weekly working hours and work intensity, resulting in bias.^11,12,13^ In addition, the lack of exploration of the relevant mechanisms is also a potential limitation, especially the consideration of behavioral lifestyles. Shift work is often associated with unhealthy lifestyles, but simply adjusting for lifestyle factors as confounders may lead to overadjustment bias, as certain lifestyle factors are likely to play a mediating role.^14,15^

Considering that shift work may be a potential risk factor for mental disorders, understanding the association of shift work with depression and anxiety, as well as the mediating role of lifestyles, may provide a basis for promoting the mental health of shift workers. Therefore, our study aimed to comprehensively investigate the associations of shift work, its type, frequency, and working years with anxiety and depression by using data from the UK Biobank, and further explore the mediating role of lifestyles between shift work and anxiety and depression.

## Methods

The UK Biobank study was approved by the North West Multi-Centre Research Ethics Committee. All participants provided informed consent through electronic signature at baseline assessment. This cohort study followed the Strengthening the Reporting of Observational Studies in Epidemiology (STROBE) reporting guideline.

### Study Design and Participants

UK Biobank is a prospective population-based cohort study that recruited more than 500 000 participants between 2006 and 2010. Each participant attended 1 of 22 assessment centers in the UK and completed a number of questionnaires. More details about UK Biobank have been reported in previous studies.^16,17^ In this cohort study, we included a total of 286 355 participants who were employed or self-employed. After excluding participants with a history of depression or anxiety at baseline and missing lifestyle information and covariates, 175 543 participants were included in the final analysis (eFigure 1 in Supplement 1).

### Exposure Assessment

In the UK Biobank, shift work is defined as a work schedule outside normal daytime working hours of 9:00 AM to 5:00 PM, which may involve working in the afternoon, evening or night, or rotating through these kinds of shifts. Night shifts are a work schedule that involves working through the normal sleeping hours, for instance, working through the hours from 12:00 AM to 6:00 AM.^18^

During the baseline survey, participants were asked to report their employment status (in paid employment or self-employed or others), years of current employment, hours worked per week, and whether the job involved shift work, night shift, walking or standing, and heavy physical labor (using 4-point Likert scales: never or rarely, sometimes, usually, or always). Our primary exposure variable was any shift work (including participants whose work sometimes, usually, or always involves shift work). Among participants involved in shift work, we further considered shift frequency (never, sometimes, usually, or always), type of shift (shift but nonnight shift workers or night shift workers), and number of years involved in shift work (years of current employment are assumed as duration of shift work).^3^

### Outcomes

We defined patients with depression or anxiety as inpatients diagnosed (primary or secondary) by physicians. Diagnoses were recorded using the *International Statistical Classification of Diseases and Related Health Problems, Tenth Revision* (*ICD-10*). Depression was defined as the first *ICD-10* record, coded F32 and F33. Anxiety was defined as *ICD-10* F40, F41, F42, and F43 (eTable 1 in Supplement 1). The diagnoses of depression and anxiety from the Hospital Episode Statistics database in England and the equivalent databases in Scotland and Wales have been validated against detailed clinical assessment and have good positive predictive value.^19,20^ The duration of follow-up was calculated from the baseline (2006-2010) to the date of death, or the date of the first depression or anxiety diagnosis, or loss to follow-up, or end of follow-up (February 28, 2018), whichever occurred first. From the baseline to the end of follow-up, participants were followed up for a median of 9.06 years.

### Measurements of Lifestyle Behaviors

Seven lifestyle behaviors, namely, physical activity, habitual diet, smoking status, drinking habit, sleep duration, sedentary time, and body mass index (BMI; calculated as weight in kilograms divided by height in meters squared) were evaluated (eTable 2 in Supplement 1). Physical activity (metabolic equivalent task [MET]-min per week) was assessed based on the MET score derived from the validated International Physical Activity Questionnaire (IPAQ) guidelines.^21^ We used national guidelines or validated thresholds to generate a dietary score that reflect the overall diet quality, including 7 components, namely, fruits, vegetables, whole grains, oily fish, refined grains, processed meat, and red meat.^22^ Healthy diet was defined as meeting 5 or more ideal diet components. Smoking status was classified as an individual who currently smoked an individual who did not smoke. Alcohol consumption was defined according to whether participants drank every day or almost every day. Sleep duration was defined as a binary variable. We used TV time as a measure of sedentary time^23^ (eTable 2 in Supplement 1).

### Covariates

We considered possible confounding, including sociodemographic factors (age, race and ethnicity, Townsend deprivation index, and educational attainment); health status (diabetes, hypertension, cancer, stroke, and ischemic heart disease [angina and heart attack]); and job-related factors (hours of work per week, years working in current job, walking or standing at work, and heavy manual or physical work).

Sociodemographic factors and job-related factors were collected through questionnaires or verbal interviews at baseline. Race and ethnicity were included because of their potential confounding or differences. Medical history was defined based on whether they had been told by a doctor that they had certain medical conditions.

### Statistical Analysis

Baseline characteristics of the participants were described by the mean (SD) of continuous variables and proportions of categorical variables by current work schedule. Analysis of variance and χ^2^ test were used to test the statistical significance of continuous and categorical variables, respectively. Kaplan-Meier curves were generated to illustrate cumulative incidence of depression and anxiety by shift work and shift frequency.

Cox proportional hazard regression models were used to calculate the hazard ratios (HRs) and 95% CIs for the associations of shift work, its type, and frequency with anxiety and depression. Several models were constructed to estimate HRs and their 95% CI. Model 0 was adjusted for age and sex only; model 1 was further adjusted for education, Townsend deprivation index, race and ethnicity, history of cancer, diabetes, hypertension, stroke and ischemic heart disease (angina and heart attack); model 2 was further adjusted for hours of work per week, years working in current job, walking or standing at work, and heavy manual or physical work. Restricted cubic splines were used to explore the dose-response association of years of shift work with depression and anxiety. Subgroup and interaction analyses were performed stratified by age, sex, socioeconomic status, heavy manual labor, and hours of work per week.

To evaluate the mediating role of lifestyles on the associations of shift work with depression and anxiety, cause mediation analyses were conducted. Cause mediation analyses were based on counterfactual framework, which formally defines both direct and indirect effects, and were more robust to the various limitations of traditional adjustment-based mediation analysis.^24^ A causal diagram of our hypothesized mediating effect and corresponding assumptions is presented in eFigure 2 in Supplement 1. Indirect, direct, and total effects were estimated by combining mediation and outcome models that adjusted for covariates in model 2 and other lifestyle factors in addition to the mediator. We used 1000 times quasi-bayesian Monte Carlo simulation to calculate the 95% CIs of the proportion mediated.

The selected lifestyle factors were considered as potential mediators in a 3-step analysis. First, we estimated the association of shift work with each lifestyle factor using the multivariate-adjusted linear or logistic regression models. Second, we used multivariate-adjusted Cox regression models to assess the associations of lifestyle factors significantly associated with shift work with the risk of depression and anxiety (eTable 3 and 4 in Supplement 1). Third, we conducted mediating analyses based on lifestyle factors that were significantly associated with work shift and depression or anxiety.^3,25^

Several sensitivity analyses were performed. First, we excluded participants who developed depression or anxiety within 1 year of recruitment to reduce the possibility of reverse causation. Second, median imputation was performed for all missing values and the main analyses were repeated using the employed population with no loss sample size (286 355 participants). Median imputation has been shown to be an effective imputation method and is not inferior to other complex methods.^26,27^ Third, we further adjusted for loneliness and social activities in the models. Fourth, we adjusted nonmediating lifestyle factors in the models to test whether the results are robust.

R version 4.0.5 (R Foundation for Statistical Computing) was used for all statistical analyses. Tests were 2-sided, and *P* < .05 was considered statistically significant. Data analysis was conducted from November 2022 to January 2023.

## Results

### Baseline Characteristic

The main sample included 175 543 adults with a mean (SD) age of 52.6 (7.1) years. Of the 175 543 employed participants, 88 290 (50.3%) were men, 167 495 (95.4%) were White adults, and 27 637 (16.2%) reported shift work. Compared with nonshift workers, participants who reported shift work status were more likely to be male, individuals from minoritized racial and ethnic groups, have higher social deprivation, lower education levels, less healthy lifestyles, more walking or standing work, and more manual labor (Table 1).

**Table 1.** Baseline Characteristics of the Study Participants According to Shift Work Status

Characteristics	Current work schedule, No. (%)		*P* value
	Nonshift workers (n = 147 906)	Shift workers (n = 27 637)	
Age, mean (SD), y	52.79 (7.08)	51.79 (6.96)	<.001
Sex			
Male	72 656 (49.12)	15 634 (56.57)	<.001
Female	75 250 (50.88)	12 003 (43.43)	
Race and ethnicity			
Black	1592 (1.08)	864 (3.13)	<.001
Chinese	320 (0.22)	85 (0.31)	
Multiple	874 (0.59)	219 (0.79)	
South Asian	2159 (1.46)	753 (2.72)	
White	142 164 (96.12)	25 331 (91.66)	
Other^a^	797 (0.54)	385 (1.39)	
Townsend deprivation index, mean (SD)	−1.70 (2.81)	−0.88 (3.15)	<.001
Educational attainment			
College or university degree	64 521 (43.62)	6705 (24.26)	<.001
A levels or AS levels or equivalent	19 247 (13.01)	3339 (12.08)	
O levels / GCSEs or equivalent	30 407 (20.56)	7138 (25.83)	
CSEs or equivalent	8415 (5.69)	2808 (10.16)	
NVQ or HND or HNC or equivalent	8982 (6.07)	2751 (9.95)	
Other professional qualifications	5638 (3.81)	1794 (6.49)	
None of the above	10 696 (7.23)	3102 (11.22)	
BMI, mean (SD)	27.00 (4.51)	27.96 (4.84)	<.001
Drink alcohol every day or almost every day	28 763 (19.40)	4097 (14.82)	<.001
Current smoking	11 759 (7.95)	3447 (12.47)	<.001
MET-min per week, mean (SD)	120.30 (97.34)	165.00 (123.93)	<.001
Unhealthy diet	108 156 (73.12)	20 941 (75.77)	<.001
Unhealthy sleep duration	35 172 (23.78)	8773 (31.74)	<.001
Unhealthy TV viewing or sedentary time	7896 (5.34)	2400 (8.68)	<.001
Years working in current job, mean (SD)	12.90 (10.71)	13.35 (10.74)	<.001
No. of work hours a week, mean (SD)	34.93 (12.47)	38.12 (12.76)	<.001
Walking or standing at work			
Never or rarely	62 019 (41.93)	4400 (15.92)	<.001
Sometimes	47 370 (32.03)	8002 (28.95)	
Usually	18 700 (12.64)	5953 (21.54)	
Always	19 817 (13.40)	9282 (33.59)	
Heavy manual or physical labor at work			
Never or rarely	110 105 (74.44)	9998 (36.17)	<.001
Sometimes	25 018 (16.91)	10 363 (37.50)	
Usually	6779 (4.58)	3806 (13.77)	
Always	6004 (4.06)	3470 (12.56)	
Medical history			
Heart disease	1686 (1.14)	366 (1.32)	<.009
Angina	1954 (1.32)	451 (1.63)	<.001
Stroke	916 (0.62)	192 (0.69)	.16
Hypertension	29 347 (19.84)	5995 (21.69)	<.001
Diabetes	4856 (3.28)	1244 (4.50)	<.001
Cancer	8360 (5.65)	1401 (5.07)	<.001

Abbreviations: A, advanced; AS, advanced subsidiary; BMI, body mass index (calculated as weight in kilograms divided by height in meters squared); CSE, Certificate of Secondary Education; GCSE, General Certificate of Secondary Education; HNC, Higher National Certificate; HND, Higher National Diploma; NVQ, National Vocational Qualification; O, ordinary; MET, metabolic equivalent of tasks.

^a^ Other includes any races or ethnicities not otherwise specified.

### Shift Work and Depression and Anxiety

**Figure 1.**
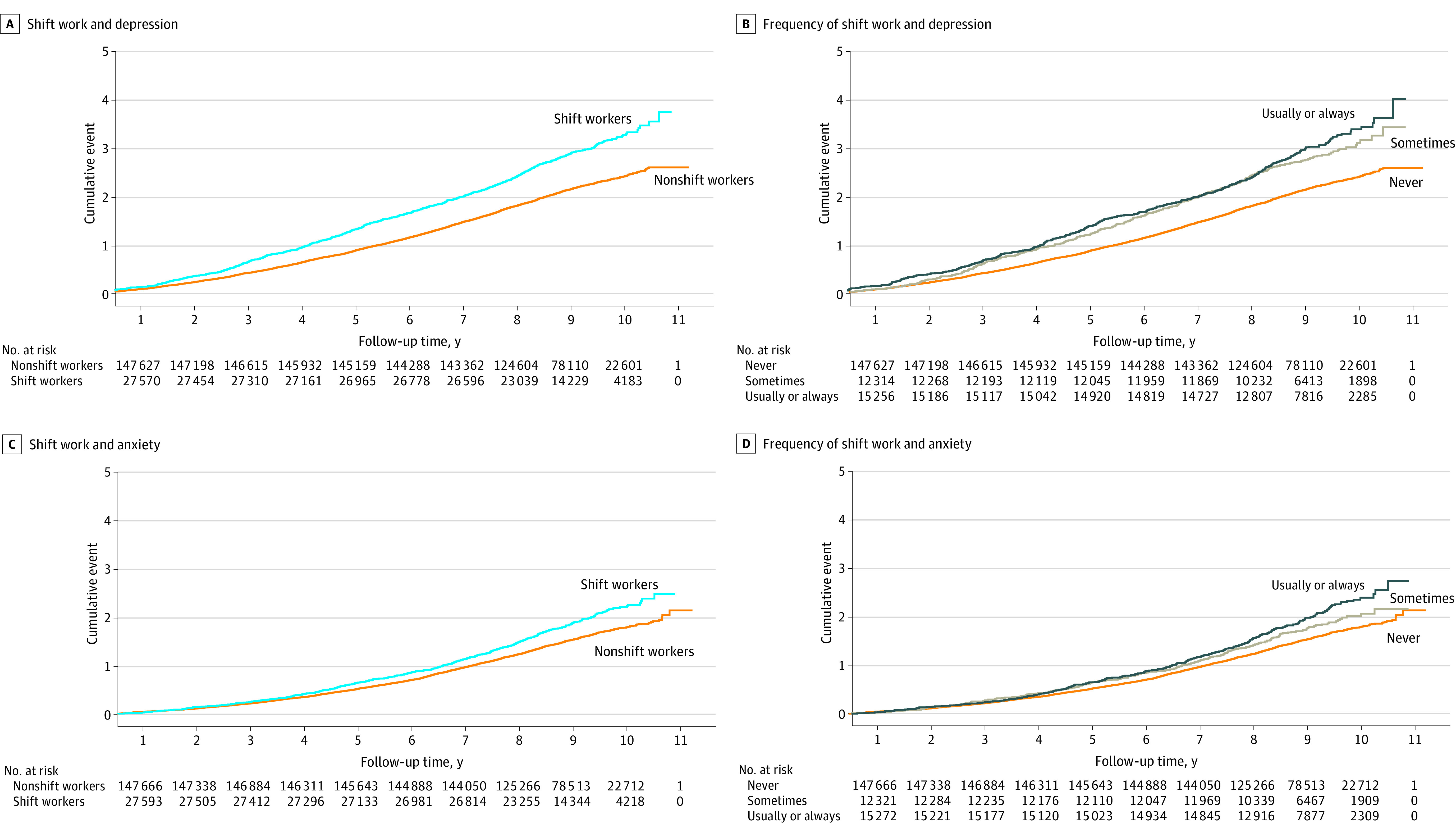
Kaplan-Meier Curves of Time to Primary Outcome of Incident Depression and Anxiety by Shift Work and Its Frequency

The Kaplan-Meier curve showed that both shift work and shift frequency were significantly associated with depression and anxiety (Figure 1). Schoenfeld residuals were used to test the proportional hazards assumption, and no violation was observed.

Table 2 showed the associations of shift work, its type, and frequency with anxiety and depression. During a median (IQR) follow-up of 9.06 (8.35-9.75) years, 3956 workers (2.3%) developed depression and 2838 (1.7%) developed anxiety. In the fully adjusted model, shift work was significantly associated with a higher risk of depression (HR, 1.22; 95% CI, 1.12-1.33; *P* < .001) and anxiety (HR, 1.16; 95% CI, 1.04-1.28; *P* < .001). Compared with workers who never worked shifts, workers who worked shifts sometimes (depression: HR, 1.23; 95% CI, 1.10-1.38; *P* < .001; anxiety: HR, 1.11; 95% CI, 0.97-1.28; *P* = .13) and usually or always (depression: HR, 1.21; 95% CI, 1.09-1.35; *P* < .001; anxiety: HR, 1.19; 95% CI, 1.05-1.35; *P* < .007) were associated with a greater risk of depression and anxiety. We did not observe a significant association of night shift work with depression and anxiety risk among shift workers (depression: HR, 1.05; 95% CI, 0.90-1.21; *P* = .55; anxiety: HR, 0.98; 95% CI, 0.82-1.17; *P* = .81).

**Table 2.** Associations of Shift Work and Its Frequency and Type With Depression and Anxiety^a^

Characteristics	Incident depression (n = 3956)		Incident anxiety (n = 2838)	
	HR (95% CI)	*P* value	HR (95% CI)	*P* value
Shift work				
Model 0				
Nonshift work	1 [Reference]	NA	1 [Reference]	NA
Shift work	1.42 (1.31-1.53)	<.001	1.30 (1.18-1.43)	<.001
Model 1				
Nonshift work	1 [Reference]	NA	1 [Reference]	NA
Shift work	1.28 (1.18-1.38)	<.001	1.19 (1.08-1.31)	<.001
Model 2				
Nonshift work	1 [Reference]		1 [Reference]	
Shift work	1.22 (1.12-1.33)	<.001	1.16 (1.04-1.28)	<.001
Physical activity	1.22 (1.13-1.33)	<.001	1.16 (1.04-1.28)	<.001
Dietary characteristics	1.22 (1.12-1.33)	<.001	1.16 (1.04-1.28)	<.001
Smoking status	1.21 (1.11-1.31)	<.001	1.14 (1.03-1.27)	<.001
Drinking	1.22 (1.12-1.33)	<.001	1.16 (1.05-1.28)	<.001
Sleep duration	1.20 (1.11-1.31)	<.001	1.14 (1.03-1.27)	<.001
TV viewing or sedentary time	1.22 (1.12-1.32)	<.001	1.15 (1.04-1.27)	<.001
BMI	1.18 (1.09-1.29)	<.001	1.14 (1.03-1.26)	<.05
Frequency of shift work				
Model 0				
Never	1 [Reference]	NA	1 [Reference]	NA
Sometimes	1.37 (1.22-1.53)	<.001	1.21 (1.05-1.39)	<.01
Usually or always	1.46 (1.32-1.61)	<.001	1.37 (1.22-1.54)	<.001
Model 1				
Never	1 [Reference]	NA	1 [Reference]	NA
Sometimes	1.27 (1.13-1.42)	<.001	1.13 (0.99-1.30)	.07
Usually or always	1.29 (1.16-1.42)	<.001	1.23 (1.09-1.39)	<.001
Model 2				
Never	1 [Reference]	NA	1 [Reference]	NA
Sometimes	1.23 (1.10-1.38)	<.001	1.11 (0.97-1.28)	.13
Usually or always	1.21 (1.09-1.35)	<.001	1.19 (1.05-1.35)	<.01
Type of shift work				
Model 0				
Shift but nonnight shift workers	1 [Reference]	NA	1 [Reference]	NA
Night shift workers	0.99 (0.86-1.14)	.88	0.92 (0.77-1.10)	.36
Model 1				
Shift but nonnight shift workers	1 [Reference]	NA	1 [Reference]	NA
Night shift workers	0.99 (0.86-1.15)	.93	0.92 (0.77-1.10)	.35
Model 2				
Shift but nonnight shift workers	1 [Reference]	NA	1 [Reference]	NA
Night shift workers	1.05 (0.90-1.21)	.55	0.98 (0.82-1.17)	.81

Abbreviations: BMI, body mass index; HR, hazard ratio; TV, television.

^a^ Model 0, age and sex only; Model 1, education, Townsend deprivation index, race and ethnicity, history of cancer, diabetes, hypertension, stroke and ischemic heart disease (angina and heart attack) additionally; Model 2, hours of work per week, years working in current job, walking or standing at work, and heavy manual or physical work additionally.

**Figure 2.**
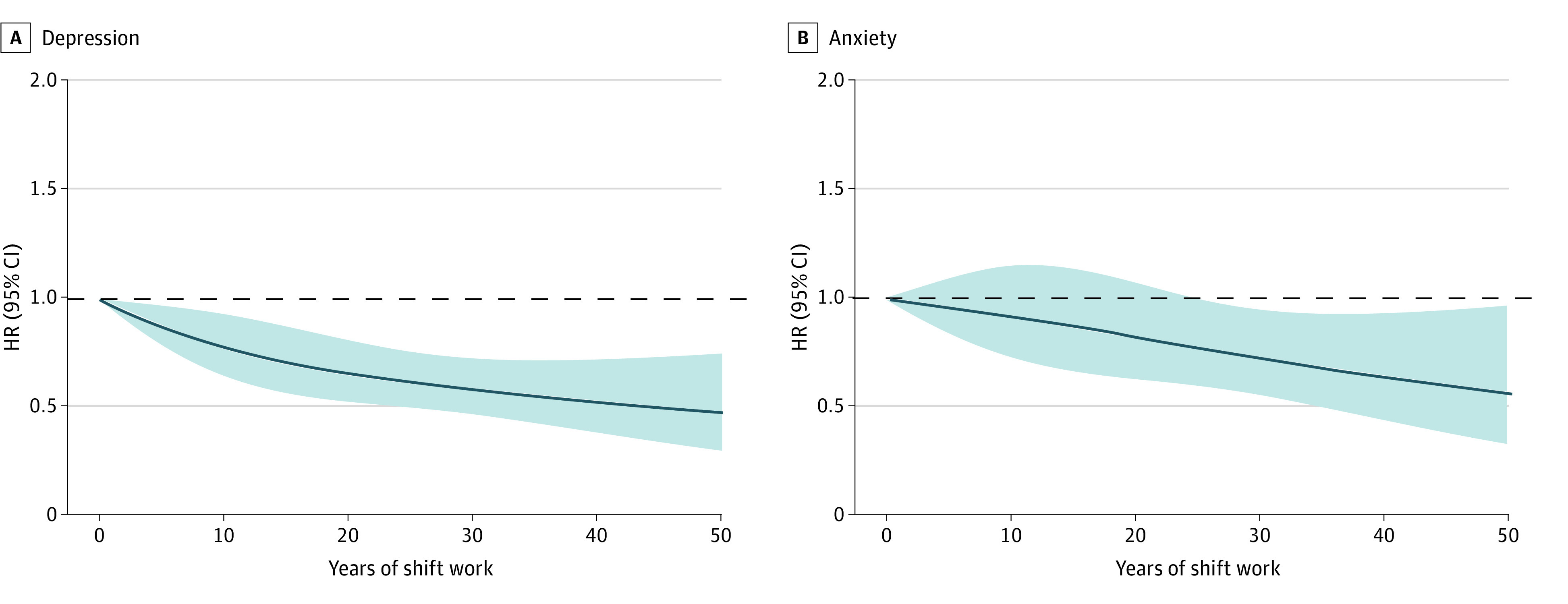
Restricted Cubic Splines of Cox Regression Showing Hazard Ratios of Primary Outcome (Incident Depression and Anxiety) by Years of Shift Work Adjusted for age, sex, education, Townsend deprivation index, race and ethnicity, history of cancer, diabetes, hypertension, stroke and ischemic heart disease (angina and heart attack), hours of work per week, walking or standing at work, and heavy manual or physical work.

In Figure 2, we used restricted cubic splines to flexibly model and visualize the dose-response association of years of shift work with depression and anxiety. We observed that years of shift work were negatively associated with the risk of depression and anxiety.

### Mediation Effects

Mediation analyses were summarized in Table 3. Lifestyle factors associated with both shift work and outcomes were selected for mediating analysis (eTable 3 and 4 in Supplement 1). We found that current smoking (7.4%; 95% CI, 4.3%-17.0%), sleep duration (7.7%; 95% CI, 4.4%-18.0%), sedentary time (1.6%; 95% CI, 0.3%-5.0%), and BMI (14.6%; 95% CI, 9.3%-30.0%) explained 31.3% of the association between shift work and depression. Similarly, current smoking (6.4%; 95% CI, 2.8%-32.0%), sleep duration (6.7%; 95% CI, 2.5%-29.0%), and BMI (8.1%; 95% CI, 3.6%-37.0%) explained 21.2% of the association of shift work with anxiety. Mediating charts containing indirect, direct and total effects of each of the mediating lifestyle factors are available in eFigures 3 and 4 in Supplement 1.

**Table 3.** Summary of Mediation Analyses

Characteristics	Association with shift work^a^	Incident depression		Incident anxiety	
		Association with outcome^b^	% Mediated (95% CI)	Association with outcome^b^	% Mediated (95% CI)
Total^c^			31.3		21.2
Physical activity (MET-min per week)	+	Ø	NA	Ø	NA
Unhealthy dietary characteristics	Ø	Ø	NA	Ø	NA
Current smoking	+	+	7.4 (4.3-17.0)	+	6.4 (2.8-32.0)
Unhealthy drinking habit	−	Ø	NA	Ø	NA
Unhealthy sleep duration	+	+	7.7 (4.4-18.0)	+	6.7 (2.5-29.0)
Unhealthy TV viewing or sedentary time	+	+	1.6 (0.3-5.0)	Ø	NA
BMI	+	+	14.6 (9.3-30.0)	+	8.1 (3.6-37.0)

Abbreviations: +, positive association; −, negative association; Ø, no significant association; BMI, body mass index (calculated as weight in kilograms divided by height in meters squared); NA, not available.

^a^ Summarized from eTable 3 in Supplement 1; multiple linear or logistic regression models with the potential mediator as the dependent variable and shift work as the independent variable. Adjusted for each other and for age, sex, education, Townsend deprivation index, race and ethnicity, history of cancer, diabetes, hypertension, stroke and ischemic heart disease (angina and heart attack), hours of work per week, years working in current job, walking/standing at work, and heavy manual/physical work.

^b^ Summarized from eTable 4 in Supplement 1; Cox regression models with potential mediators as independent variables. Adjusted for each other and for shift work, age, sex, education, Townsend deprivation index, race and ethnicity, history of cancer, diabetes, hypertension, stroke and ischemic heart disease (angina and heart attack), hours of work per week, years working in current job, walking or standing at work, and heavy manual or physical work.

^c^ Total percentages mediated were the sum of percentages from all significant mediators (all mediators being mutually adjusted in analyses).

### Subgroup, Interaction, and Sensitivity Analyses

As shown in eTable 5 in Supplement 1, the associations of shift work with depression and anxiety did not differ among age, sex, socioeconomic status, and hours of work per week, except for heavy manual labor (depression: HR for never or rarely, 1.47; 95% CI, 1.30-1.66; HR for sometimes or more, 1.08; 95% CI, 0.96-1.20; anxiety: HR for never or rarely, 1.38; 95% CI, 1.19-1.60; HR for sometimes or more, 1.01; 95% CI, 0.88-1.16; all P for interaction < .001). The associations between shift work and depression and anxiety were more pronounced in participants with minimal heavy manual labor work (depression: HR, 1.47; 95% CI, 1.30-1.66; *P* < .001; anxiety: HR, 1.38; 95% CI, 1.19-1.60; *P* < .001) than in participants with more heavy manual labor work (depression: HR, 1.08; 95% CI, 0.96-1.20; *P* = .20; anxiety: HR, 1.01; 95% CI, 0.88-1.16; *P* = .88). When we excluded participants who developed depression or anxiety within 1 year of recruitment, used the median imputation, further adjusted for loneliness and social activities, or adjusted nonmediating lifestyle factors in the model, our results remained robust (eTable 6 in Supplement 1).

## Discussion

In this cohort study of 175 543 participants, we found that shift work was significantly associated with a higher risk of depression and anxiety and that the risk was positively associated with shift frequency. However, there was no significant difference between night shifts and nonnight shifts among shift workers. In the dose-association analyses, years of shift work were negatively associated with the risk of depression and anxiety. By testing the mediation effect, smoking, sedentary time, BMI, and sleep duration were found to partially mediate the association between shift work and depression and anxiety.

Our main findings are consistent with existing evidence. A meta-analysis involving 28 431 participants showed that shift workers were at greater risk for poor mental health, especially depressive symptoms.^10^ A longitudinal study from the British Household Panel Survey reported that shift workers were more likely to have depression or anxiety, especially female shift workers.^28^ Building on these studies, our study identified the association between shift work and depression and anxiety in a larger sample population, and further clarified the role of shift frequency, shift type, and shift years. Interestingly, our analysis showed a significant negative association between years of shift work and risk of depression and anxiety. Adjustment to the job may be an explanation. Fatigue tends to increase rapidly at the beginning of shift work, leading to poor mental health, but over time (after adaptation to shift work), increased proficiency on the job reduces fatigue and leads to recovery of mental health.^29,30,31^ In addition, we found no difference in the risk of depression and anxiety between night shifts and nonnight shifts, which is inconsistent with previous finding.^8^ Definitions of night shift in different countries or research contexts may provide an explanation. For example, night shifts in the Netherlands generally commence between 10:00 PM and 12:00 AM, which is different from the definition in the UK Biobank, leading to the difference in results.^31^ Further studies in different cultural contexts are necessary to confirm our findings.

Although the harm of shift work depends on a variety of factors (eg, socioeconomic status, access to social resources), it is generally considered to be significantly associated with behavioral lifestyles, as shift work is often associated with low income, poor environment, and subjective stress, which are more likely to lead to unhealthy lifestyles.^15,32^ Therefore, in order to examine the pathways associated with shift work causing depression and anxiety, we analyzed the mediating role of various lifestyle factors. Our results suggested that the associations of shift work with anxiety and depression may be partly explained by smoking, sleep duration, and BMI and that the risk of depression may additionally be explained by sedentary time. If causal, intervention on these modifiable lifestyles could reduce the incidence of depression and anxiety in shift workers. Our mediation analyses may contribute to a better understanding of the underlying mechanisms between shift work and depression and anxiety and may provide valuable insights for promoting health equity among shift workers in the context of accelerating globalization.

### Strengths and Limitations

To the best of our knowledge, the current study is the largest study of the associations and pathways between shift work and anxiety and depression. Our study covered the general population across occupations, making up for the limitations of previous occupation-specific cohorts. In addition, we have further clarified the roles of multiple lifestyle factors (confounding or mediating) and the relative importance of mediating lifestyle factors, helping to guide and improve strategy development and practice in health areas related to shift work.

This study had limitations. First, although we adjusted for as many confounders as possible, the impact of residual confounders (eg, genetic factors, unmeasured diseases) could not be completely eliminated. Second, the loss of covariate information led to a loss of sample size, which may understate the association between shift work and risk of depression and anxiety. However, our results remained robust when we performed median imputation for all missing values and repeated the main analysis using the employed population (286 355 participants) with no lost sample size. Third, we measured only baseline occupational and lifestyle information, and the associations of long-term trajectories of occupational status with depression and anxiety could not be examined. Future research should give priority to life course methods.

## Conclusions

In this cohort study, we found that shift work was associated with higher risk for depression and anxiety, especially when individuals were newly involved in shift work. Our study not only supports that shift work should be considered an occupational hazard, but also provides evidence for the urgent need for the development of public health interventions that promote healthy lifestyles aimed at improving the mental health of shift workers.

## Notes

Supplement 1. **eTable 1.** Ascertainment for Depression and Anxiety Cases in the UK Biobank Study **eTable 2.** Assessment of Lifestyle Factors **eTable 3.** Associations Between Shift Work and Potential Mediators **eTable 4.** Association Between All Potential Mediators and Depression and Anxiety and Mediation Proportions **eTable 5.** Associations of Shift Work With Depression and Anxiety by Subgroup **eTable 6.** Sensitive Analyses of Shift Work and Its Frequency and Type in Relation to Depression and Anxiety **eFigure 1.** Flowchart for the Selection of the Study Population **eFigure 2.** Causal Diagram for the Association Between Shift Work (X), Lifestyle Factors (M), and Depression or Anxiety (Y) **eFigure 3.** Mediation Effects of Lifestyle Factors on the Association of Shift Work and Depression **eFigure 4.** Mediation Effects of Lifestyle Factors on the Association of Shift Work and Anxiety

Supplement 2.
Data Sharing Statement
