# Supplementary material for: Lifestyle Factors in the Association of Shift Work and Depression and Anxiety
Source: JAMA Netw Open. 2023 Aug 14;6(8):e2328798. doi: 10.1001/jamanetworkopen.2023.28798 (PMC10425829; doi:10.1001/jamanetworkopen.2023.28798)
Supplement: Supplement 1. — eTable 1. Ascertainment for Depression and Anxiety Cases in the UK Biobank Study eTable 2. Assessment of Lifestyle Factors eTable 3. Associations Between Shift Work and Potential Mediators eTable 4. Association Between All Potential Mediators and Depression and Anxiety and Mediation Proportions eTable 5. Associations of Shift Work With Depression and Anxiety by Subgroup eTable 6. Sensitive Analyses of Shift Work and Its Frequency and Type in Relation to Depression and Anxiety eFigure 1. Flowchart for the Selection of the Study Population eFigure 2. Causal Diagram for the Association Between Shift Work (X), Lifestyle Factors (M), and Depression or Anxiety (Y) eFigure 3. Mediation Effects of Lifestyle Factors on the Association of Shift Work and Depression eFigure 4. Mediation Effects of Lifestyle Factors on the Association of Shift Work and Anxiety [file jamanetwopen-e2328798-s001.pdf]

Supplemental Online Content

Xu M, Yin X, Gong Y. Lifestyle factors in the association of shift work and depression and anxiety. *JAMA Netw Open*. 2023;6(8):e2328798.
doi:10.1001/jamanetworkopen.2023.28798

eTable 1. Ascertainment for Depression and Anxiety Cases in the UK Biobank Study
eTable 2. Assessment of Lifestyle Factors
eTable 3. Associations Between Shift Work and Potential Mediators
eTable 4. Association Between All Potential Mediators and Depression and Anxiety and Mediation Proportions
eTable 5. Associations of Shift Work With Depression and Anxiety by Subgroup
eTable 6. Sensitive Analyses of Shift Work and Its Frequency and Type in Relation to Depression and Anxiety
eFigure 1. Flowchart for the Selection of the Study Population
eFigure 2. Causal Diagram for the Relationship Between Shift Work (X), Lifestyle Factors (M), and Depression or Anxiety (Y)
eFigure 3. Mediation Effects of Lifestyle Factors on the Association of Shift Work and Depression
eFigure 4. Mediation Effects of Lifestyle Factors on the Association of Shift Work and Anxiety

This supplemental material has been provided by the authors to give readers additional information about their work.

© 2023 Xu M et al. *JAMA Network Open.*

eTable 1. Ascertainment for depression and anxiety cases in the UK Biobank study

	**ICD-10**
Depression	F32.0, F32.1, F32.2, F32.3, F32.8, F32.9, F33.0, F33.1, F33.2, F33.3, F33.4, F33.8, F33.9
Anxiety	F40.0, F40.1, F40.2, F40.8, F40.9, F41.0, F41.1, F41.2, F41.3, F41.8, F41.9, F42.0, F42.1, F42.2, F42.8, F42.9, F43.0, F43.1, F43.2, F43.8, F43.9

© 2023 Xu M et al. *JAMA Network Open.*

eTable 2. Assessment of lifestyle factors

UK Biobank			
**Lifestyle factor**	**Questionnaire**	**Healthy habit**	**Unhealthy habit**
Smoking status	"Do you smoke tobacco now?" and "In the past, how often have you smoked tobacco?"	past or never smoker	current
Alcohol intake	"About how often do you drink alcohol?"	≤ 4 times week	Daily or almost daily
Physical activity	International Physical Activity Questionnaire short form – total time walking or moderate or vigorous-intensity physical activity	Expressed as a continuous variable (Metabolic Equivalent Task (MET)-min per week)	
Sleep duration	"About how many hours sleep do you get in every 24 hours?"	≥7 or ≤9h/day	<7 or >9h/day
BMI	BMI was calculated by dividing weight (in kilograms) by the square of height (in metres).	Expressed as a continuous variable	
TV viewing / sedentary time	"In a typical day, how many hours do you spend watching TV?"	< 4 h/day	≥ 4 h/day
Diet score (From Touchscreen questionnaire at baseline. Healthy diet was defined as meeting five or more ideal diet components)			
Fruit	"About how many of …. would you eat per day?" Separate questions for pieces of fresh and dried fruit.	≥3 servings/day	< 3 servings/day
Vegetable intake	"About how many of …. would you eat per day?" Separate questions for tablespoons of salad or cooked/raw vegetables.	≥3 servings/day	< 3 servings/day
Oily fish intake	"How often do you eat oily fish? (e.g. sardines, salmon, mackerel, herring)"	≥2 servings/week	< 2 servings/week
Red meat intake	"How often do you eat…?" Separate questions for Beef / lamb or mutton / pork (excluding processed meats such as ham or bacon).	≤1.5 servings/week	>1.5 servings/week
Processed meat intake	"How often do you eat processed meats (such as bacon, ham, sausages, meat pies, kebabs, burgers, chicken nuggets)?"	≤1 servings/week	>1 servings/week
whole grains	"How many slices of bread do you eat each WEEK?" and "What type of bread do you mainly eat?"; "How many bowls of cereal do you eat a WEEK?" and "What type of cereal do you mainly eat?" (brown bread, wholemeal and wholegrain bread, bran and oat cereal were defined as whole grains)	≥3 servings/day	< 3servings/day
Refined grains	"How many slices of bread do you eat each WEEK?" and "What type of bread do you mainly eat?"; "How many bowls of cereal do you eat a WEEK?" and "What type of cereal do you mainly eat?" (white and other type of bread, biscuit cereal, muesli and other type of cereal were defined as refined grains)	≤1.5 servings/day	> 1.5 servings/day

© 2023 Xu M et al. *JAMA Network Open.*

eTable 3. Associations between shift work and potential mediators

	β (95% CI)	*P* value
Physical activity (MET-min per week)	3.70 (2.49-4.90)	< 0.0001
Unhealthy dietary characteristics*	-0.008 (-0.040-0.025)	0.65
Current smoker*	0.21 (0.16-0.25)	< 0.0001
Unhealthy drinking habit*	-0.13 (-0.17-0.09)	< 0.0001
Unhealthy sleep duration*	0.22 (0.19-0.25)	< 0.0001
Unhealthy TV viewing / sedentary time*	0.21 (0.16-0.27)	< 0.0001
BMI	0.51 (0.45-0.57)	< 0.0001

β: standardised regression coefficients; MET: metabolic equivalent of tasks; TV: television; BMI, body mass index;
* binary variables modelled by logistic regressions; the exponentiation of βs are odds ratios;
Adjusted for each other and for shift work, age, sex, education, Townsend deprivation index, ethnicity, history of cancer, diabetes, hypertension, stroke and ischemic heart disease (angina and heart attack), hours of work per week, years working in current job, walking/standing at work, and heavy manual/physical work.

© 2023 Xu M et al. *JAMA Network Open.*

eTable 4. Association between all potential mediators and depression and anxiety and mediation proportions

	Incident depression			Incident anxiety		
	Outcome regressed by potential mediator	Mediation analysis		Outcome regressed by potential mediator	Mediation analysis	
	HR (95% CI)	% mediated	$P_{mediation}$	HR (95% CI)	% mediated	$P_{mediation}$
Physical activity (MET-min per week)	1.00 (0.99-1.01)	-	-	1.00 (0.99-1.01)	-	-
Unhealthy dietary characteristics*	1.02 (0.95-1.09)	-	-	1.01 (0.93-1.10)	-	-
Current smoker*	1.70 (1.55-1.86)	7.4	< 0.0001	1.48 (1.32-1.65)	6.4	0.02
Unhealthy drinking habit*	1.02 (0.94-1.12)	-	-	1.09 (0.98-1.20)	-	-
Unhealthy sleep duration*	1.30 (1.21-1.39)	7.7	< 0.0001	1.21 (1.12-1.31)	6.7	0.03
Unhealthy TV viewing / sedentary time*	1.16 (1.03-1.29)	1.6	0.02	1.10 (0.96-1.27)	-	-
BMI	1.05 (1.04-1.06)	14.6	< 0.0001	1.02 (1.01-1.03)	8.1	0.02

HR: hazard ratio; CI: confidence interval; MET: metabolic equivalent of tasks; TV: television; BMI, body mass index;
* binary variables modelled by logistic regressions;
Adjusted for each other and for shift work, age, sex, education, Townsend deprivation index, ethnicity, history of cancer, diabetes, hypertension, stroke and ischemic heart disease (angina and heart attack), hours of work per week, years working in current job, walking/standing at work, and heavy manual/physical work.

© 2023 Xu M et al. *JAMA Network Open.*

eTable 5. Associations of shift work with depression and anxiety by subgroup

	Incident depression		Incident anxiety	
	HR (95% CI)	$P_{interaction}$	HR (95% CI)	$P_{interaction}$
Age		0.48		0.40
≤ 50	1.19 (1.04-1.35)		1.15 (0.98-1.34)	
> 50	1.25 (1.12-1.39)		1.14 (1.00-1.31)	
Sex		0.16		0.29
Female	1.25 (1.12-1.39)		1.17 (1.02-1.33)	
Male	1.18 (1.03-1.35)		1.15 (0.98-1.35)	
Socioeconomic status (Townsend deprivation index)		0.41		0.24
More deprived	1.26 (1.14-1.40)		1.22 (1.07-1.39)	
Less deprived	1.17 (1.02-1.35)		1.07 (0.91-1.26)	
Heavy manual labour		< 0.01		< 0.01
Never or rarely	1.47 (1.30-1.66)		1.38 (1.19-1.60)	
Sometimes or more	1.08 (0.96-1.20)		1.01 (0.88-1.16)	
Hours of work per week (using the median as the cut-off point)		0.07		0.52
≤ 35	1.29 (1.13-1.46)		1.17 (1.00-1.36)	
> 35	1.18 (1.05-1.31)		1.14 (1.00-1.31)	

HR: hazard ratio; CI: confidence interval;
Adjusted for age, sex, education, Townsend deprivation index, ethnicity, history of cancer, diabetes, hypertension, stroke and ischemic heart disease (angina and heart attack), hours of work per week, years working in current job, walking/standing at work, and heavy manual/physical work. Each group adjusted for the other covariates except itself.

© 2023 Xu M et al. *JAMA Network Open.*

eTable 6. Sensitive analyses of shift work and its frequency and type in relation to depression and anxiety

	Incident depression		Incident anxiety	
	HR (95% CI)	*P* value	HR (95% CI)	*P* value
**Sensitive 1**				
Shift work				
Non-shift work	Ref.		Ref.	
Shift work	1.22 (1.12-1.33)	< 0.0001	1.17 (1.06-1.30)	< 0.01
Frequency of shift work				
Never	Ref.		Ref.	
Sometimes	1.24 (1.10-1.39)	< 0.001	1.13 (0.98-1.31)	0.09
Usually / Always	1.20 (1.08-1.34)	< 0.001	1.20 (1.06-1.37)	< 0.01
Type of shift work				
Shift but non-night shift workers	Ref.		Ref.	
Night shift workers	1.07 (0.92-1.24)	> 0.05	0.97 (0.81-1.16)	> 0.05
**Sensitive 2**				
Shift work				
Non-shift work	Ref.		Ref.	
Shift work	1.27 (1.19-1.34)	< 0.0001	1.14 (1.06-1.23)	< 0.001
Frequency of shift work				
Never	Ref.		Ref.	
Sometimes	1.23 (1.13-1.34)	< 0.0001	1.11 (1.00-1.23)	< 0.05
Usually / Always	1.29 (1.20-1.39)	< 0.0001	1.16 (1.06-1.28)	< 0.01
Type of shift work				
Shift but non-night shift workers	Ref.		Ref.	
Night shift workers	1.05 (0.95-1.16)	> 0.05	1.00 (0.88-1.14)	> 0.05
**Sensitive 3**				
Shift work				
Non-shift work	Ref.		Ref.	
Shift work	1.18 (1.08-1.28)	< 0.001	1.13 (1.02-1.26)	< 0.05
Frequency of shift work				
Never	Ref.		Ref.	
Sometimes	1.19 (1.06-1.34)	< 0.01	1.11 (0.96-1.28)	> 0.05
Usually / Always	1.16 (1.04-1.30)	< 0.01	1.15 (1.01-1.32)	< 0.05

© 2023 Xu M et al. *JAMA Network Open.*

Type of shift work				
Shift but non-night shift workers	Ref.		Ref.	
Night shift workers	1.10 (0.95-1.28)	> 0.05	0.98 (0.82-1.18)	> 0.05
**Sensitive 4**				
Shift work				
Non-shift work	Ref.		Ref.	
Shift work	1.22 (1.13-1.33)	< 0.01	1.15 (1.04-1.28)	< 0.01
Frequency of shift work				
Never	Ref.		Ref.	
Sometimes	1.24 (1.10-1.39)	< 0.0001	1.11 (0.97-1.28)	> 0.05
Usually / Always	1.21 (1.09-1.35)	< 0.01	1.19 (1.05-1.35)	< 0.01
Type of shift work				
Shift but non-night shift workers	Ref.		Ref.	
Night shift workers	1.04 (0.90-1.21)	> 0.05	0.98 (0.82-1.17)	> 0.05

HR: hazard ratio; CI: confidence interval;
Adjusted for age, sex, education, Townsend deprivation index, ethnicity, history of cancer, diabetes, hypertension, stroke and ischemic heart disease (angina and heart attack), hours of work per week, years working in current job, walking/standing at work, and heavy manual/physical work.
Sensitive 1: Excluding participants with incident depression or anxiety < 1 year from baseline.
Sensitive 2: For primary outcome associated with current work schedule in the imputed dataset.
Sensitive 3: Further adjust for loneliness and social activities. The definition of loneliness was based on the question "Do you often feel lonely?"; the definition of leisure activities was based on the question "Which of the following do you attend once a week or more often"? (Sports club or gym, Pub or social club, Religious group, Adult education class, and other group activity).
Sensitive 4: Further adjustment for non-mediating lifestyle factors (physical activity, dietary characteristics, and drinking habit for depression; physical activity, dietary characteristics, sedentary time, and drinking habit for anxiety).

© 2023 Xu M et al. *JAMA Network Open.*

eFigure 1. Flowchart for the selection of the study population

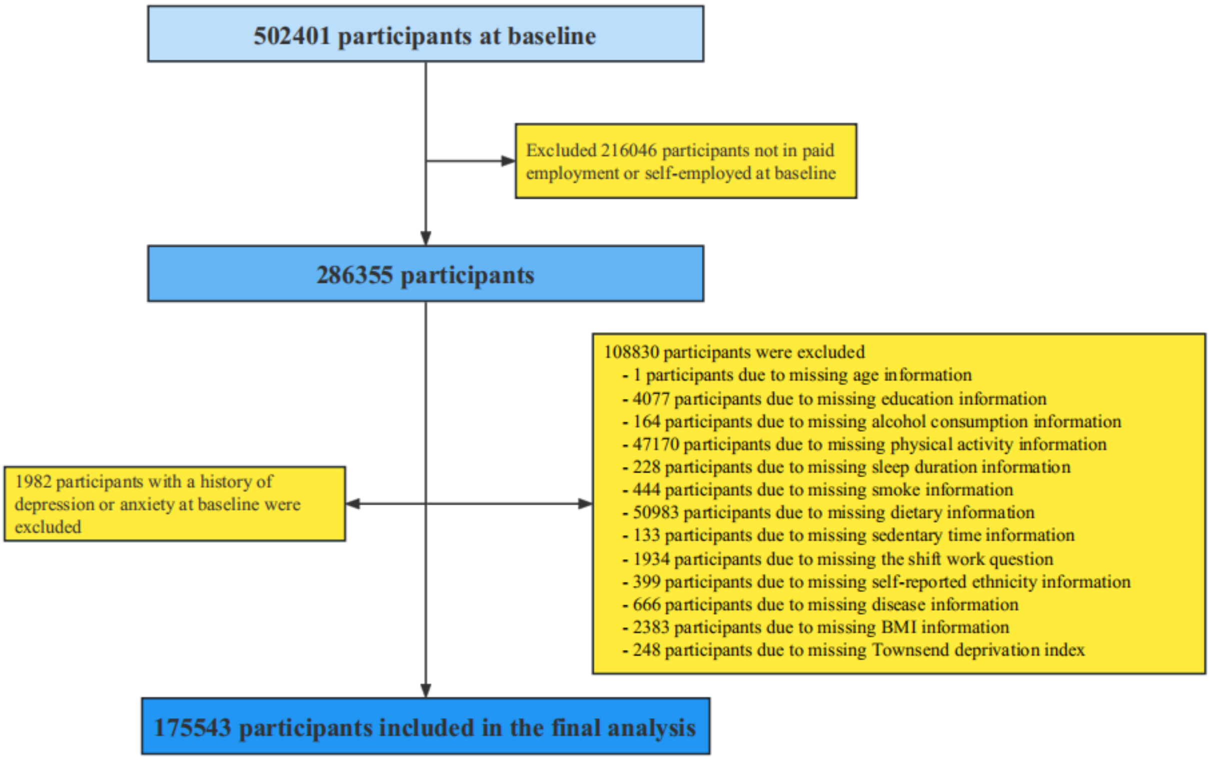

© 2023 Xu M et al. *JAMA Network Open.*

eFigure 2. Causal diagram for the relationship between shift work (X), lifestyle factors (M), and depression or anxiety (Y).

C and its solid arrows indicate measured confounders (age, sex, education, Townsend deprivation index, ethnicity, history of cancer, diabetes, hypertension, stroke and ischemic heart disease (angina and heart attack), hours of work per week, years working in current job, walking/standing at work, and heavy manual/physical work), U_{xy} indicates unmeasured confounders of X-Y relation, U_{my} indicates unmeasured confounders of M-Y relation, U_{xm} indicates unmeasured confounders of X-M relation, U_c indicates no measured or unmeasured confounders of the M-Y relation affected by X conditional on C.

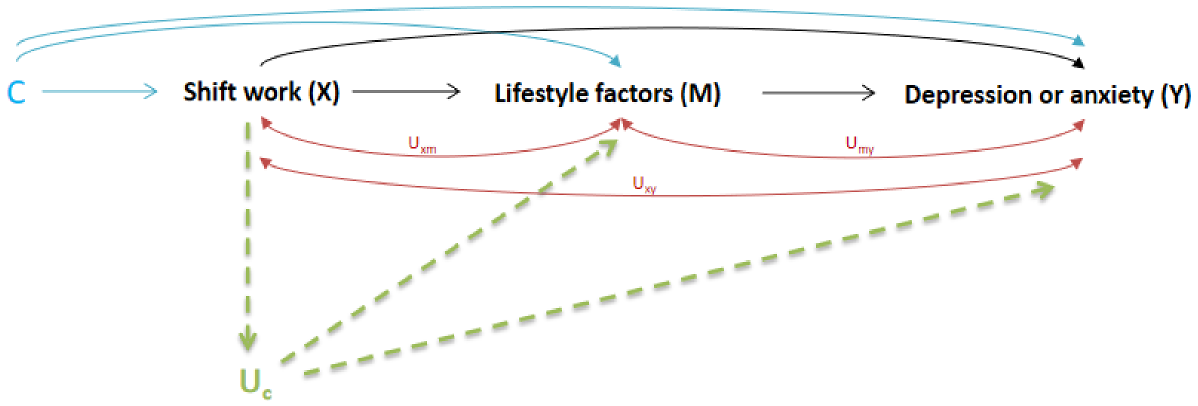

© 2023 Xu M et al. *JAMA Network Open.*

eFigure 3. Mediation effects of lifestyle factors on the association of shift work and depression.

ADE, average direct effects; ACME,

average causal mediation effects. Note: The mediation analysis was carried out by “mediation” package in R, with 1000 simulations

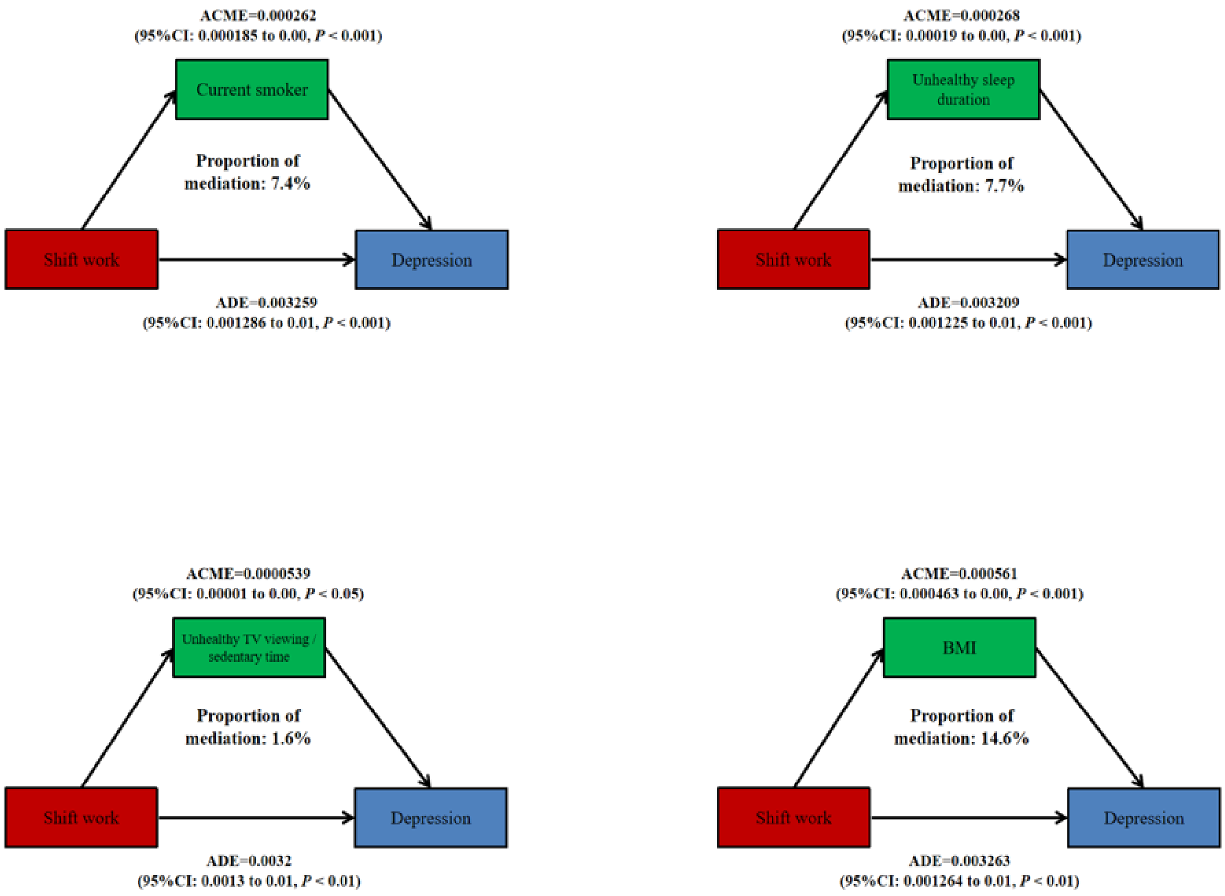

© 2023 Xu M et al. *JAMA Network Open.*

eFigure 4. Mediation effects of lifestyle factors on the association of shift work and anxiety.

ADE, average direct effects; ACME,

average causal mediation effects. Note: The mediation analysis was carried out by “mediation” package in R, with 1000 simulations

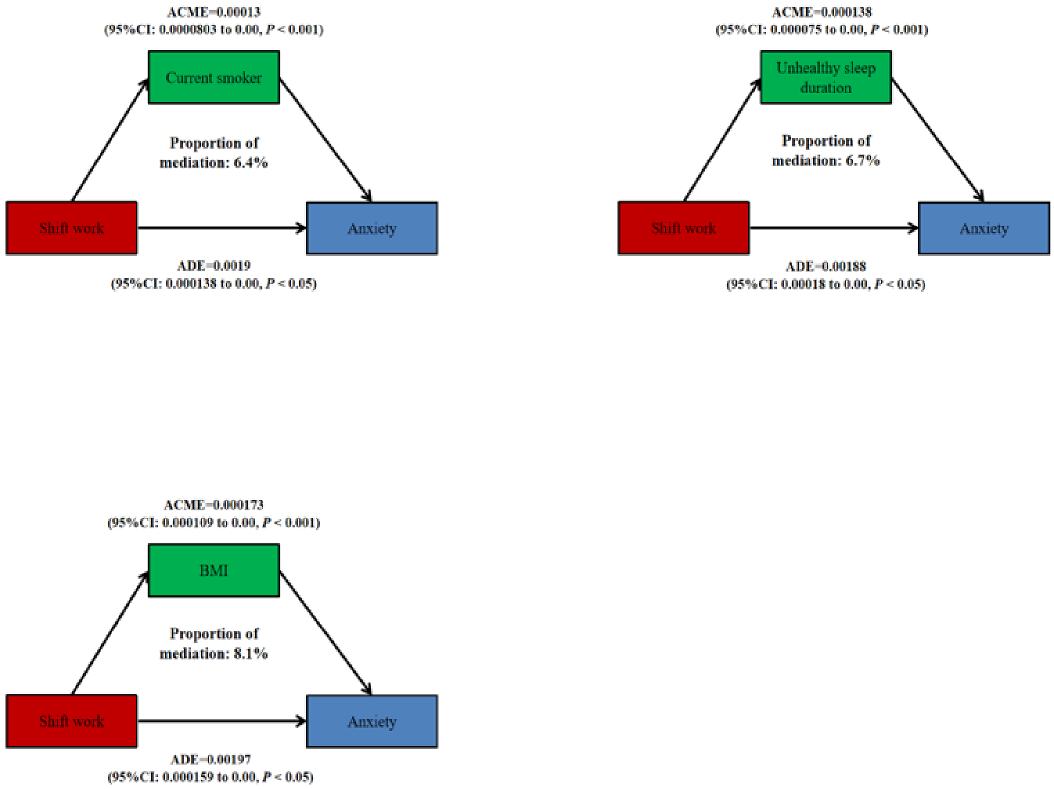

© 2023 Xu M et al. *JAMA Network Open.*
